# Comparison of Black Hole Sign, Satellite Sign, and Iodine Sign to Predict Hematoma Expansion in Patients with Spontaneous Intracerebral Hemorrhage

**DOI:** 10.1155/2021/3919710

**Published:** 2021-02-01

**Authors:** Milind Ratna Shakya, Fan Fu, Miao Zhang, Yi Shan, Fan Yu, Shengjun Sun, Jie Lu

**Affiliations:** ^1^Department of Radiology, Xuanwu Hospital, Capital Medical University, No. 45 Changchun Street, Xicheng District, Beijing, China; ^2^Beijing Key Laboratory of Magnetic Resonance Imaging and Brain Informatics, Xuanwu Hospital, Capital Medical University, No. 45 Changchun Street, Xicheng District, Beijing, China; ^3^Neuroradiology Department, Beijing Neurosurgical Institute, Beijing Tiantan Hospital, Capital Medical University, No. 119 Nansihuanxilu, Fengtai District, Beijing, China; ^4^Department of Nuclear Medicine, Xuanwu Hospital, Capital Medical University, No. 45 Changchun Street, Xicheng District, Beijing, China

## Abstract

**Purpose:**

To discretely and collectively compare black hole sign (BHS) and satellite sign (SS) with recently introduced gemstone spectral imaging-based iodine sign (IS) for predicting hematoma expansion (HE) in spontaneous intracerebral hemorrhage (SICH).

**Methods:**

This retrospective study includes 90 patients from 2017 to 2019 who underwent both spectral computed tomography angiography (CTA) as well as noncontrast computed tomography (NCCT) within 6 hours of SICH onset along with subsequent follow-up NCCT scanned within 24 hours. We named the presence of any of BHS or SS as any NCCT sign. Two independent reviewers analyzed all the HE predicting signs. Receiver-operator characteristic curve analysis and logistic regression were performed to compare the predictive performance of HE.

**Results:**

A total of 61 patients had HE, out of which IS was seen in 78.7% (48/61) while BHS and SS were seen in 47.5% (29/61) and 41% (25/61), respectively. The area under the curve for BHS, SS, and IS was 63.4%, 67%, and 82.4%, respectively, while for any NCCT sign was 71.5%. There was no significant difference between IS and any NCCT sign (*P* = 0.108). Multivariate analysis showed IS (odds ratio 68.24; 95% CI 11.76-396.00; *P* < 0.001) and any NCCT sign (odds ratio 19.49; 95% CI 3.99-95.25; *P* < 0.001) were independent predictors of HE whereas BHS (odds ratio 0.34; 95% CI 0.01-38.50; *P* = 0.534) and SS (odds ratio 4.54; 95% CI 0.54-38.50; *P* = 0.165) had no significance.

**Conclusion:**

The predictive accuracy of any NCCT sign was better than that of sole BHS and SS. Both any NCCT sign and IS were independent predictors of HE. Although IS had higher predictive accuracy, any NCCT sign may still be regarded as a fair predictor of HE when CTA is not available.

## 1. Introduction

Spontaneous intracerebral hemorrhage (SICH) accounts for 10% to 30% of all strokes worldwide [1]. It is one of the most devastating types of stroke, leading to neurological deterioration and case fatality. Owing to its high morbidity and mortality rate, the prediction of early hematoma expansion (HE) is essential [2]. Generally, early HE is seen in up to 20% to 30% of patients with SICH [3]. However, previous literature has shown up to 73% of patients exert some degree of HE [4–6]. Therefore, reliable imaging tools are required for predicting HE and providing appropriate treatment.

Based on hematoma shape and density, numerous noncontrast computed tomography- (NCCT-) based HE predicting markers are available [7, 8]. We enrolled one HE predictor from each of shape and density group. The black hole sign (BHS) attributes density heterogeneity trait, whereas the satellite sign (SS) attributes shape irregularity traits. A computed tomography angiography (CTA) spot sign is the robust marker for HE prediction. In correspondence with spot sign, both BHS and SS are proven to be the independent predictors for HE with a significant association [9–12]. The advantage of BHS and SS are its evident visibility in NCCT, which is often available in a clinical setting. Furthermore, it is beneficial in patients with chronic kidney conditions and in patients who are allergic to contrast medium.

Recently, gemstone spectral imaging- (GSI-) based iodine sign (IS) has also been introduced as a reliable and sensitive marker for predicting HE [13]. As an assuring scan technique, GSI can efficiently differentiate iodine from the blood product. During active bleeding, its monochromatic imaging can quantitatively measure the concentration of iodine leaking from the bleeding site. A threshold of iodine concentration greater than 7.82 (100 *μ*g/ml) has a significant association with HE [14–16]. However, the correlation between GSI-based IS with NCCT-based predicting markers is yet to be known.

Since the predictive efficiency of BHS and SS has not been tallied with GSI-based IS, this study's objective is to compare the predictive performance of NCCT-based BHS and SS with GSI-based IS for prediction of HE in patients with SICH. Besides, we also compared the predictive capability of any NCCT sign against IS.

## 2. Materials and Methods

### 2.1. Study Population

We retrospectively studied 90 consecutive cases of SICH patients above the age of 18 years. All patients enrolled in this study were admitted to Beijing Tiantan Hospital between November 2017 and December 2019. Informed consent for all the cases was obtained either from the patients or from next to kin. The inclusion criteria for this study were (1) patients who underwent NCCT as well as contrast-enhanced dual-energy spectral computed tomography angiography (CTA) within 6 hours after onset of symptoms and (2) follow-up NCCT was scanned within 24 hours after the initial scan. Patients were excluded from the study if they had SICH history or had secondary SICH caused by an arteriovenous malformation, brain tumor, traumatic brain injury, hemorrhagic transformation of ischemic infarction, moyamoya disease, or anticoagulant induced SICH. Patients were also excluded if the initial spectral CTA was not done due to chronic kidney conditions. Patients could not undergo a follow-up CT scan due to emergency surgical intervention of hematoma evacuation, transferred to another center, or was pronounced dead. The clinical, baseline demography and radiological variables of all the patients, along with hypertensive history, diabetic history, smoking history, alcohol abuse, glucose baseline, the National Institutes of Health Stroke Scale (NIHSS) scores, the duration form the onset of the symptoms to initial CT scan time, and lab results, were collected at the emergency department by neurologists.

### 2.2. Image Acquisition

We used the 64-slice discovery CT750HD scanner (GE Healthcare, Waukesha, WI) to scan all the patients. Initial NCCT was done by standard single-energy helical mode with 120 kVp, 300 mA, 0.6 s gantry rotation time, and 0.984 : 1 helical pitch. Subsequent contrast-enhanced CTA was done using dual-energy spectral imaging mode with rapid switching between 80 kVp and 140 kVp. Scanning parameters were arranged as tube current, 375 mA; slice thickness, 5 mm; rotation time, 0.6 s/rotation; collimation thickness, 0.625 mm × 64; and scan field-of-view, 25 cm. Both the reconstruction slice thickness and interval were 0.625 mm. The contrast agent Iohexol (300 mg I/ml; Guerbet, France) was used in the spectral CTA scans according to a patient weight-dependent dose of 0.7 ml/kg and an injection rate of 6 ml/s. Time from contrast bolus injection to CTA acquisition was obtained from archived SmartPrep images (GE Healthcare). Final follow-up plain CT was obtained within 24 hours using the same CT system and parameters.

### 2.3. Imaging Analysis

**Figure 1:**
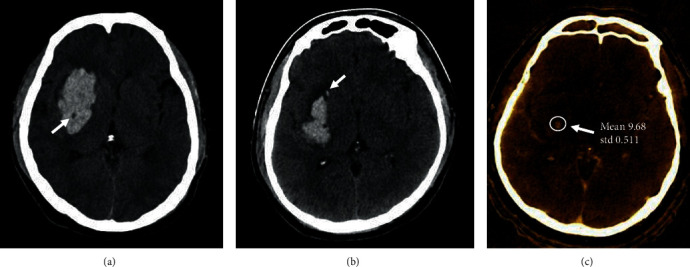
An illustration of hematoma expansion predicting signs. (a) Axial noncontrast computed tomography (NCCT) illustrates black hole sign described as the hypoattenuated area encapsulated within the area of hyperattenuation, with a difference of at least 28 Hounsfield units between two densities. (b) Axial NCCT shows a satellite sign described as the presence of small hemorrhage, which is completely detached from the main hemorrhage, observed in at least a single-CT slice. (c) Axial plane on iodine-based decomposition represents an iodine sign with a tiny enhanced focus whose iodine concentration is 9.68 100 *μ*g/ml (>7.82 100 *μ*g/ml).

Firstly, two independent reviewers blinded to the patients' clinical data evaluated all the NCCT images for the presence or absence of BHS and SS from the initial scan. The BHS (Figure 1(a)) was defined as the hypoattenuated area of a round, oval, or a rod-like shape encapsulated within the hyperattenuated area, which is not connected to the adjacent brain tissue, with the difference of at least 28 Hounsfield units between two densities [9]. The SS (Figure 1(b)) was defined as a small hemorrhage of maximum transverse diameter, not more than 10 mm, which is completely detached from the main hemorrhage seen in at least a single CT slice. The distance between the main hemorrhage and the detached small hemorrhage should not be more than 1-20 mm. Subarachnoid and intraventricular hemorrhages were not counted as SS [11]. We named the presence of any of the BHS or the SS as any NCCT sign. The presence of any NCCT sign was noted.

Secondly, the reviewers evaluated positive or negative IS from the spectral CTA images. The IS was extracted with GSI viewer on standard advantage workstation (AW 4.6; GE Healthcare) of dual-energy spectral CT. Positive IS (Figure 1(c)) was defined as the presence of tiny enhancement of ≥1 foci within hematoma with an iodine concentration of >7.82 (100 *μ*g/ml) inside the foci, visualized on iodine-based decomposition image [13, 14].

Hematoma volume of initial and follow-up CT scans was measured on separate occasions; the images were randomized to blind the raters from patients' information. The location of hematoma was evaluated and was categorized as either deep or lobar. The presence or absence of midline shift was noted. Intraventricular hematoma extension was noted but was not included in the volumetric analysis of HE. Hematoma volume of initial and follow-up CT images was manually calculated using the ABC/2 formula. HE was defined as an absolute growth of hematoma by at least 6 ml (follow-up volume–initial volume), or a relative growth by at least 33% (absolute growth/initial volume) [4, 17].

### 2.4. Statistical Analysis

All the statistical analyses for this study were performed using SPSS version 26.0 and NCSS 2019 version 19.0.3 statistical software. The categorical variables were expressed as absolute values (percentages, %), and the continuous variables were expressed as medians (IQR) or mean ± standard deviation (SD) according to the distribution of data. An independent-samples *t*-test was used for continuous variables, while Pearson chi-square or Fisher exact test was used for categorical variables in order to verify the analysis of statistical significance.

Receiver-operating characteristic (ROC) curve analysis was performed to check the diagnostic accuracy of all the predictive markers for HE. The *Z* test was performed to compare the area under the ROC of IS and any NCCT signs. Sensitivity, specificity, positive predictive value, and negative predictive value of all predictive markers were calculated. The association between hematoma expansion and all its predictive markers was evaluated using univariate and multivariate logistical regression module. The result of the regression module was presented as odds ratio (OR) and 95% confidence intervals (CI). Adjusted variables for multivariate analysis were selected. A two-tailed *P* value of <0.05 was considered statistically significant for all tests. Cohen's kappa analysis was performed for interreviewer reliability.

## 3. Results

### 3.1. Baseline Characteristics

A total of 90 patients were included in this study. The mean age of the patients was 53.88 ± 12.89 years, among which 62 (68.9%) were male, and 28 (31.1%) were female. Out of the patients studied, 61 (67.8%) of the patients showed HE. Hematomas located in deep and lobar were 72 (80%) and 18 (20%), respectively. The mean initial hematoma volume was 19.69 ± 17.02 ml. Patients with HE was more likely to have higher admission NIHSS score with *P* = 0.015. On the other hand, no significant differences in the distribution of age, gender, medical history, glucose level, midline shift, the onset of symptoms to initial CT scan duration, and intraventricular hemorrhage were observed (Table 1).

### 3.2. Interreviewer Agreement and Predictive Performance of NCCT Signs and Iodine Sign

In this study, BHS and SS were present in 35 (38.9%) and 27 (30%) patients, respectively; among them, 47.5% of positive BHS had HE (*P* = 0.02) and 41% of positive SS had HE (*P* = 0.001). On the contrary, IS was found in 52 (57.8%) patients, where 78.7% had a HE (*P* < 0.001) (Table 1). Interreviewer agreement for the detection of BHS, SS, and IS between the two reviewers was as follows BHS (*κ* = 0.89), SS (*κ* = 0.88), and IS (*κ* = 0.92).

**Figure 2:**
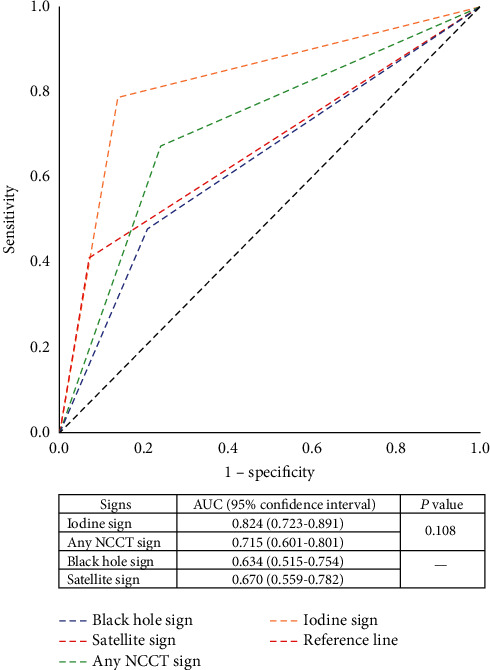
Receiver-operator characteristic curves analysis of black hole sign (BHS), satellite sign (SS), iodine sign (IS), and any noncontrast computed tomography (NCCT) sign for hematoma expansion prediction. Graph represents the area under the curve (AUC) of BHS = 63.4%, SS = 67%, IS = 82.4%, and any NCCT sign = 71.5% (island sign vs. any NCCT sign; *P* = 0.108).

The area under the curve (AUC) (Figure 2) of BHS and SS were 63.4% and 67%, respectively. The comparison between the AUC of IS and the AUC of any NCCT sign was 82.4% and 71.5%, respectively, which showed no significant difference (*P* = 0.108). The predictive performance of IS showed the highest sensitivity with 78.7%, while SS showed the highest specificity (93.1%) and the highest PPV (92.6%). Accuracy of both BHS and SS was 57.8%, while any NCCT sign showed increased accuracy of 70%. However, IS maintained its highest level of accuracy of 81.1% (Table 2).

### 3.3. Relationship between BHS, SS, Any NCCT Sign, and IS with HE

Logistic regression was performed to assess the relationship between clinical and radiological parameters with HE. In univariable logistic regression, excluding age (*P* = 0.160), fibrinogen (*P* = 0.083), activated partial prothrombin time (APTT) (*P* = 0.064), and initial hematoma volume (IHV) (*P* = 0.088), other variables like NIHSS score (OR 1.12; 95% CI 1.02-1.23; *P* = 0.019), BHS (OR 3.47; 95% CI 1.24-9.74; *P* = 0.018), SS (OR 9.38; 95% CI 2.04-3.05; *P* = 0.004), IS (OR 23.08; 95% CI 6.81-78.20; *P* < 0.001), and presence of any NCCT sign (OR 6.44; 95% CI 2.36-17.59; *P* < 0.001) were associated with HE (Table 3).

In multivariate logistic regression (Table 3), the utilized adjusted variables were age, initial hematoma volume, and NIHSS score according to a literature review. While all the adjusted values like age, initial hematoma volume, and NIHSS score showed no relation with HE (*P* > 0.05), the presence of BHS (*P* = 0.534) and SS (*P* = 0.165) also showed no relation. On the contrary, IS (OR 68.24; 95% CI 11.76-396; *P* < 0.001) and presence of any of the NCCT sign (OR 19.49; 95% CI 3.99-95.25; *P* < 0.001) were independent predictors for HE.

## 4. Discussion

**Figure 3:**
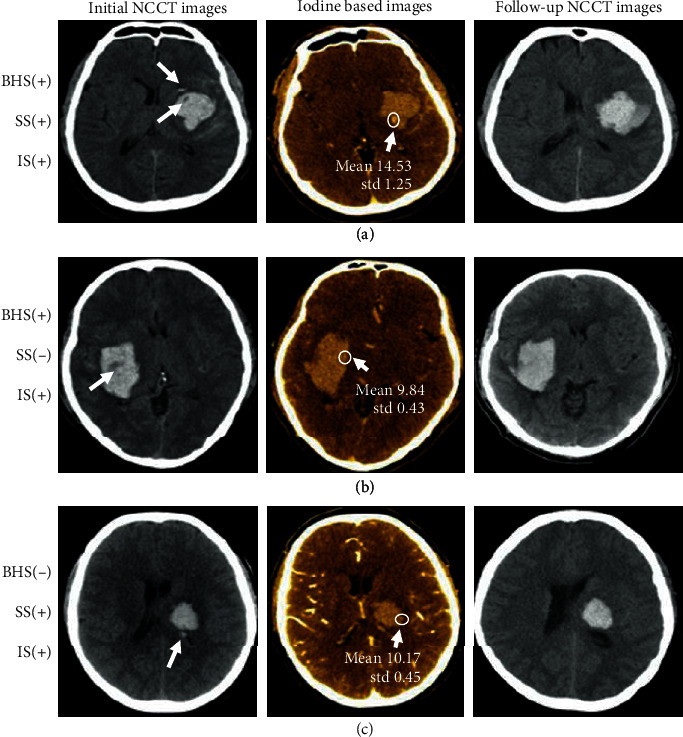
Axial images of three different patients with spontaneous intracerebral hemorrhage. (a) Initial noncontrast computed tomography (NCCT) illustrates both black hole sign (BHS) and satellite sign (SS), with iodine concentration (IC) of 14.53 (100 *μ*g/ml) on the iodine-based scan. Follow-up NCCT shows hematoma expansion (HE) of 10.3 ml. (b) The initial NCCT image illustrates positive BHS, with an IC of 9.84 (100 *μ*g/ml) on the iodine-based scan. Follow-up NCCT shows HE of 12.6 ml. (c) Initial NCCT represents a positive SS, with IC of 10.17 (100 *μ*g/ml) on the iodine-based scan. Follow-up NCCT presented with HE of 8.7 ml.

This is the first study to correlate the predicting capability of GSI-based IS with NCCT-based BHS and SS for the prediction of HE (Figure 3). The SS displayed the highest specificity and PPV in predicting HE, while the IS had the largest AUC. Moreover, the presence of any NCCT signs, i.e., BHS or SS, was better in predicting HE than the presence of a single NCCT sign. Although GSI-based IS had a higher predictive accuracy than any NCCT sign, no statistical significance was found (*P* = 0.108). Hence, any NCCT sign is still an acceptable predictor for HE when CTA is unavailable. We demonstrated that the presence of any NCCT sign and IS were independently associated with HE.

Generally, HE was observed in up to 19% to 38% of patients with SICH. However, in the phase II trial to confirm HE reduction by recombinant activated factor VII, 70% of patients with a HE has been reported [4, 6]. Furthermore, at 1-hour duration after the initial CT scan, 33% of HE were reported, while the addition of 12% of substantial HE could be observed in 1-hour to 20-hour duration [5]. This implies HE proportion may occasionally rise with time. At the same time, our study shows that 67.8% of patients of SICH had HE. Our analysis also showed (Table 1) parameter like the NIHSS score (*P* = 0.015) had a statistical significance. Previous studies suggest that this parameter is associated HE [17, 18].

In prior studies, it has been demonstrated that there is an association of hematoma heterogeneity and HE [19]. The BHS as a novel sign to determine heterogeneity for the evaluation of HE was proposed by Li et al. [9] in 2016. It had low sensitivity (31.9%) and high specificity (94.1%), which is similar to our result. The low sensitivity may be due to the small sample size of HE with positive BHS. Pathologically, the hypoattenuated region in NCCT indicates the presence of fresh blood, and the hyperattenuated region indicates the blood serum that has been isolated out of the hematoma after clot formation. Thus, positive BHS suggests the ejection of blood over a period of time [9, 20].

Additionally, the hematoma irregularity was also independently associated with hemorrhage growth and poor functional outcome [8, 21, 22]. The SS was primarily discovered by Shimoda et al. [11], which is a shape-based indicator for predicting HE, and it was said to be present mostly in hematomas with irregular shapes. However, the relationship between shape irregularity and HE was contradicted. According to a study by Barras et al. [7], it was not found to be an independent predictor for HE. Correspondingly, Takeda et al.'s [23] study also failed to identify the significant association between shape irregularity and HE, while the irregular shape of hematoma was found to be independently associated with HE in Blacquiere et al.'s [24] study. The contradiction between studies may be due to the unclear definition of irregular shapes, and a higher degree of irregularity may increase the chances of hematoma growth. Later, Yu et al. [12] suggested that the SS as an independent predictor of HE, and a recent study by Deng et al. [25] revealed the predictive accuracy of the SS of 62% while our study showed relatively lower predictive accuracy (57.8%).

Fu et al. [13] first reported the IS as a novel predictor of HE, which was regarded as early contrast extravasation on ongoing bleeding of hematoma in neighboring microvessels [26, 27], although expansion-prone hematoma [21], described as the presence of at least one of BHS, blend sign, or island sign, showed good predicting value (OR 28.33; 95% CI 12.95-61.98). However, a specific scanning technique of GSI could efficiently separate iodine and blood via a rapid kVp switching method. During active blood loss, iodine product leaks from the punctured vessel. GSI monochromatic imaging can reflect iodine concentration form the leak point [15, 16]. Moreover, IS showed higher sensitivity (91.5%) and accuracy (85.7%), which was even higher than that of the previously reckoned biomarker for HE prediction, the spot sign [13]. However, since CTA is contraindicated in certain conditions and is not readily available in every facility, NCCT-based predicting signs are essential. Our study not only compared the predictive capability of individual SS and BHS with IS but also compared the presence of any of BHS or SS with IS. Including BHS or SS collectively not just increased their previously low sensitivity (47.5% BHS; 41% SS) to 78.7% but also increased their low accuracy (57.8% BHS; 57.8% SS) to 70%.

While comparing IS with any NCCT sign, IS demonstrated higher sensitivity and specificity. Moreover, IS had a larger AUC than any NCCT sign, but no significant difference was observed (*P* = 0.108). Both, IS and any NCCT sign appeared to be an independent predictor for HE. IS appears as a better predictor for HE in SICH, but any NCCT signs may also be regarded as a reliable predictor for HE.

Estimating HE depends on numerous clinical, radiological, medical, and laboratory aspects. The treatment strategy in reducing HE might be an intensive lowering of increased admission time blood pressure. An ultraearly regime of the blood-pressure-lowering module depends on nicardipine administration within 2 hours of the symptom to reduce HE and improve outcomes [28]. Previous studies involving intensive blood pressure reduction regime, radiologic HE predicting NCCT, and CTA-spot sign have failed to delineate beneficial results. Most studies even stated that the chances of HE is optimal in the first hour after the onset [29, 30]. Correspondingly, Qi et al. [28] stated that the possibility of predicting HE by these radiologic markers overshadowed the powerful effect of the time to intervention, which might also be in the case of GSI-based IS. The prospect of radiologic and therapeutic intervention within 2 hours of onset is possible, but the practicality is questionable in a larger cohort.

Various limitations should be taken into consideration while interpreting our results. It is a single center-based retrospective study with minimal sample size. Potential selection bias may be true since only scans performed within 6 hours were studied. Furthermore, we compared only two of the NCCT-based HE predicting signs with GSI-based IS, though we accept the vicinity of other NCCT signs that predicts HE. Therefore, further longitudinal validation with large sample size and multicentred prospective study in the future is required to compare the clinical scenario rightfully.

## 5. Conclusion

Our study demonstrates that the presence of GSI-based IS and the presence of any NCCT sign are independent predictors of HE. GSI-based IS has a better predictive value for HE with higher sensitivity and accuracy, but the presence of any NCCT sign may also be regarded as a reliable predictor. Furthermore, BHS and SS are fair predictors and may be considered in settings where spectral imaging is not available or contraindicated.

## Figures and Tables

**Table 1:** Baseline demographic, clinical, and radiological comparison of patients with and without hematoma expansion.

	Entire study (*n* = 90)	Hematoma expansion (*n* = 61)	No hematoma expansion (*n* = 29)	*P* value
Age, years, mean ± SD	53.88 ± 12.89	52.56 ± 12.85	56.66 ± 12.75	0.160
Gender, male (%)	62 (68.9)	43 (70.5)	19 (65.5)	0.634
Medical history				
Hypertension (%)	73 (81.1)	50 (82)	23 (79.3)	0.763
Diabetes mellitus (%)	17 (18.9)	11 (18)	6 (20.7)	0.779
Smoking (%)	56 (62.2)	41 (67.2)	15 (51.7)	0.171
Alcohol consumption (%)	65 (72.2)	45 (73.8)	20 (69)	0.802
Clinical parameters				
Glucose, mmol/L, median (IQR)	6.40 (5.49-7.18)	6.40 (5.50-7.30)	6.40 (5.47-6.70)	0.725
Fibrinogen, mean ± SD	2.57 ± 0.606	2.49 ± 0.623	2.73 ± 0.544	0.078
APTT(s), median (IQR)	26.50 (24.30-29.00)	27.10 (25.10-29.50)	25.60 (24.10-27.20)	0.059
INR, median (IQR)	0.93 (0.89-0.98)	0.94 (0.88-0.98)	0.93 (0.89-0.96)	0.450
NIHSS, median (IQR)	9 (5.75-13)	10 (6-14)	7 (5-10)	0.015
Radiological data				
Location, deep (%)	72 (80)	50 (82)	22 (75.9)	0.499
IHV, ml, mean ± SD	19.69 ± 17.02	21.85 ± 18.57	15.17 ± 12.26	0.082
IVH, present (%)	24 (26.7)	14 (23)	10 (34.5)	0.248
Midline shift, present (%)	10 (11.1)	7 (11.5)	3 (10.3)	0.873
Onset to CT, hours, median (IQR)	2 (1.5-3.5)	2 (1.5-3.5)	2 (1.5-3.5)	0.782
Black hole signs, present (%)	35 (38.9)	29 (47.5)	6 (17.1)	0.02
Satellite sign, present (%)	27 (30)	25 (41)	2 (6.9)	0.001
Iodine sign, present (%)	52 (57.8)	48 (78.7)	4 (13.8)	<0.001
Any NCCT sign, present (%)	61 (67.8)	41 (67.21)	20 (68.97)	<0.001

IQR: interquartile range; SD: standard deviation; APTT: activated partial prothrombin time; INR: international normalized ratio; NIHSS: National Institutes of Health Stroke Scale; IHV: initial hematoma volume; IVH: intraventricular hemorrhage; NCCT: noncontrast computed tomography.

**Table 2:** Predictive performance of hematoma predictors.

	Sensitivity (%)	Specificity (%)	PPV (%)	NPV (%)	Accuracy (%)
Black hole sign	47.5	79.3	82.9	41.8	57.8
Satellite sign	41	93.1	92.6	42.9	57.8
Iodine sign	78.7	86.2	92.3	65.8	81.1
Any NCCT sign	67.2	75.9	85.4	52.4	70

PPV: positive predictive value; NPV: negative predictive value.

**Table 3:** Univariate and multivariate analysis for hematoma expansion predictors.

Variables	Univariate		Multivariate	
	OR (95% CI)	*P* value	OR (95% CI)	*P* value
Age	0.98 (0.94–1.07)	0.160	0.97 (0.92-1.03)	0.300
Initial hematoma volume	1.03 (0.99–1.06)	0.088	0.96 (0.92-1.00)	0.051
NIHSS	1.12 (1.02–1.23)	0.019	1.13 (0.97-1.33)	0.129
Black hole sign	3.47 (1.24-9.74)	0.018	0.34(0.01-10.23)	0.534
Satellite sign	9.38 (2.04-3.05)	0.004	4.54(0.54-38.50)	0.165
Iodine sign	23.08 (6.81–78.20)	<0.001	68.24 (11.76–396.00)	<0.001
Any NCCT sign	6.44 (2.36–17.59)	<0.001	19.49 (3.99–95.25)	<0.001

OR: odds ratio; CI: confidence interval; NIHSS: National Institutes of Health Stroke Scale; NCCT: noncontrast computed tomography.

## Data Availability

The data used to support the findings of this study are available from the corresponding authors upon request.
